# Progress, Advantages, and Challenges of Topological Material Catalysts

**DOI:** 10.1002/smsc.202100106

**Published:** 2022-02-03

**Authors:** Ruikuan Xie, Tan Zhang, Hongming Weng, Guo-Liang Chai

**Affiliations:** ^1^ State Key Laboratory of Structural Chemistry Fujian Institute of Research on the Structure of Matter Chinese Academy of Sciences Fuzhou Fujian 350002 P. R. China; ^2^ University of Chinese Academy of Sciences Beijing 100190 P. R. China; ^3^ Beijing National Research Center for Condensed Matter Physics Institute of Physics Chinese Academy of Sciences Beijing 100190 P. R. China

**Keywords:** catalysts, topological materials, topological surface states

## Abstract

Topological materials is one of the hottest topics in condensed matter physics because of its exotic properties such as robust metallic boundary states, Fermi arcs, and the spin‐momentum‐locking helicity. The topologically protected conducting boundary states spanning the whole bandgap are expected to serve as robust and wide‐range‐energy transition states facilitating catalytic reactions. Recently, some topological materials have been found to be high‐performance catalysts, which might open an emerging research field. Herein, an overview of topological materials is given and then recent progress in topological material catalysts (TMCs) is presented. As it is a new field, more detailed and accurate mechanisms behind the high performance of TMCs are urgently needed. Combining theoretical and experimental studies is a promising way to resolve these puzzles. Heterostructures, dopants, and defects have the chance to tune the catalytic activity of TMCs while retaining topological surface states (TSSs). Also, more TMCs are needed to be discovered, and more catalytic reactions are to be investigated for TMCs in the future.

## Graphical abstract

Topological material catalyst (TMC) has shown exciting potential which might open an emerging research field. The progress, advantages, and challenges for TMC are presented in this Perspective. The main challenge is that the mechanism behind high performance is unclear. Topological surface state is supposed to be the key factor and possible methods to investigate it are put forward.

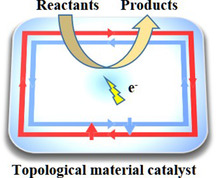

## 1 Brief Introduction of Topological Materials

Topological materials have attracted great attention since the theoretical prediction and experimental realization of topological insulators.^[^ ^1^, ^2^, ^3^, ^4^ ^]^ Different types of topological materials have been discovered in recent years, both in insulators and metals. In this section, a brief introduction of each kind of topological material is given and their exotic properties and potential applications are discussed.

**Figure 1:**
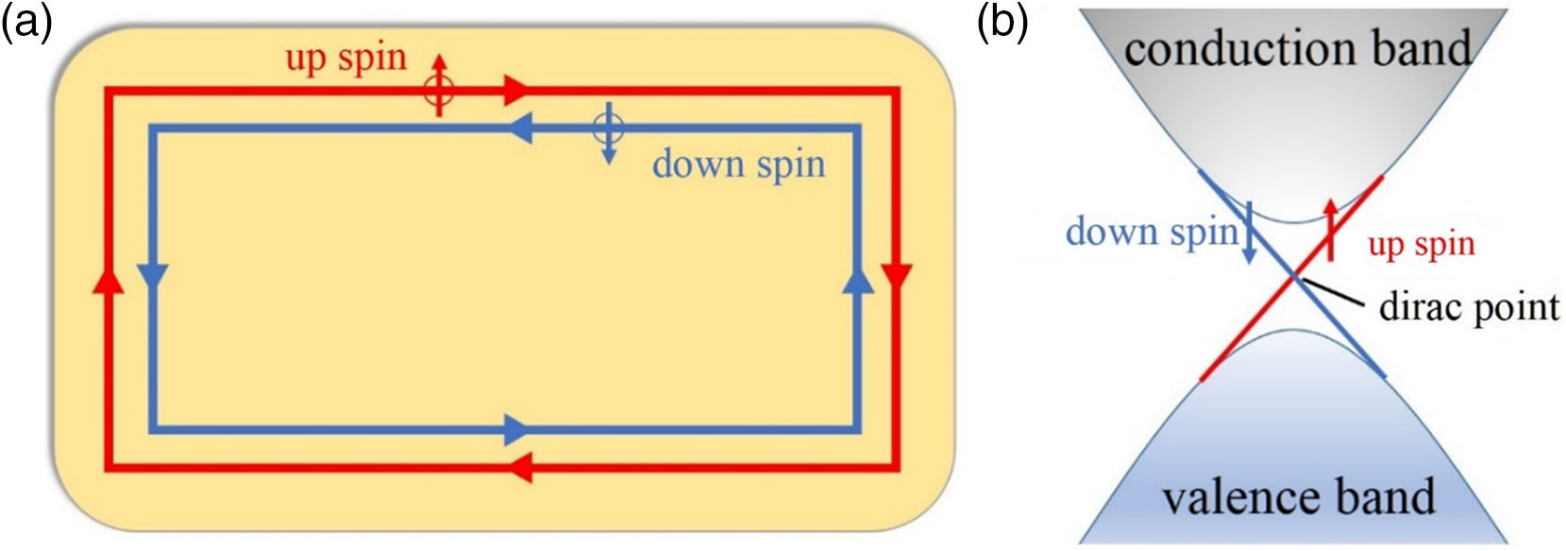
a) The schematic of metallic edge states of a 2D topological insulator, in which spin‐up and spin‐down electrons counterpropagate. b) The idealized spin‐resolved band structure of the edge states corresponding to (a) forming a 1D Dirac cone.

Based on band theory, materials can be divided into two categories considering their electrical conductivity: insulators and conductors. Materials with band structures where the valence bands are completely filled with electrons and separated from the conduction bands with a bandgap are called insulators or semiconductors. While for conductors valence bands or conduction bands are partially occupied and there is no bandgap, topological insulators have bulk energy bandgap like ordinary insulators, but they are featured with a gapless edge or surface states for 2D or 3D topological insulators, respectively. This conducting boundary state is topologically nontrivial and robust as protected by bulk band topology. Take 2D topological insulators for instance, two edge states with opposite spin polarizations counterpropagate due to spin‐momentum‐locking effect, as shown in **Figure** **1**a. The edge/surface states present linear Dirac‐cone‐like dispersions (Figure 1b), which make the physics of relativistic Dirac fermion relevant. Electron transport in topological edge/surface states (TSSs) are protected from backscattering even with crystal disorder, which allows them to be dissipationless. TSSs span over the whole bandgap and they are inherently robust against boundary modification from defects, impurity, and adsorbent. Topological insulators have been experimentally observed in many materials, such as 2D HgTe/CdTe quantum well and 3D Bi_2_Se_3_ family compounds.^[^ ^1^, ^4^, ^5^, ^6^ ^]^ Many other exotic phenomena are also involved, such as linear magnetoresistance,^[^ ^7^, ^8^ ^]^ Shubnikov‐de Hass oscillations,^[^ ^9^, ^10^ ^]^ Aharonov−Bohm effect, extremely high carrier mobility,^[^ ^11^, ^12^ ^]^ and so on.

**Figure 2:**
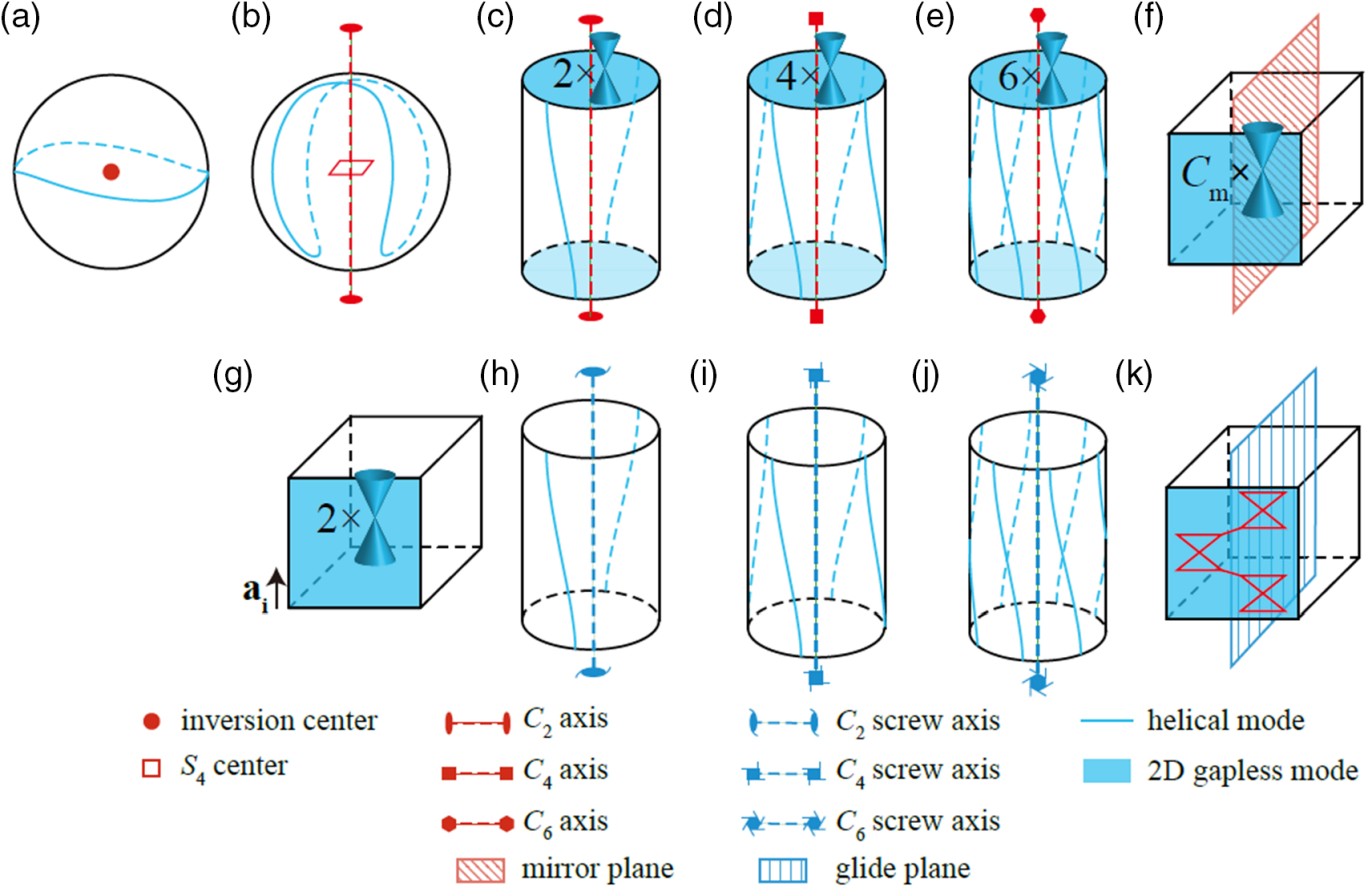
Surface states of TCIs. a) The surface state of inversion‐protected TCI. b)The surface state of S_4_‐protected TCI. c–e) The surface states of *C*_*n* = 2;4;6_‐rotation‐protected TCI. f) The surface state of mirror TCI. g) The surface state of weak topological insulator; here, *a*_*i*_ represents the translation symmetry protecting the weak index. h–j) The surface states of *C*_*n* = 2;4;6_‐screw‐protected TCI. k) The surface state of the glide mirror‐protected TCI. Reproduced under the terms of the CC‐BY 4.0 license.^[^ ^18^ ^]^ Copyright 2018, The Authors, published by Springer Nature.

In addition to the earlier topological insulators protected by time‐reversal symmetry,^[^ ^13^, ^14^, ^15^ ^]^ symmetry‐protected topological classification was extended to crystalline symmetry by Fu and this type of topological insulator is named topological crystalline insulator (TCI).^[^ ^16^ ^]^ The first theoretically predicted and experimentally confirmed TCI SnTe has a simple rock salt structure and its TSSs with even number of Dirac cones can be observed on the surfaces, respecting the mirror symmetry protecting the nonzero mirror Chern number.^[^ ^17^ ^]^ Besides the mirror symmetry, the inversion, rotation, screw, and glide symmetries can also protect nontrivial topological invariants (as illustrated in **Figure** **2**)^[^ ^18^, ^19^, ^20^, ^21^ ^]^ and even lead to high‐order topological insulators.^[^ ^22^ ^]^ High‐order topological insulators can host conducting topological states in their boundaries of lower than (*n*−2) dimension, such as the hinge states (1D) or coner states (0D) in 3D or 2D high‐order topological insulators. Obviously, these topological boundary states are highly sample shape dependent as the crystalline symmetry protecting the topology should be conserved.

**Figure 3:**
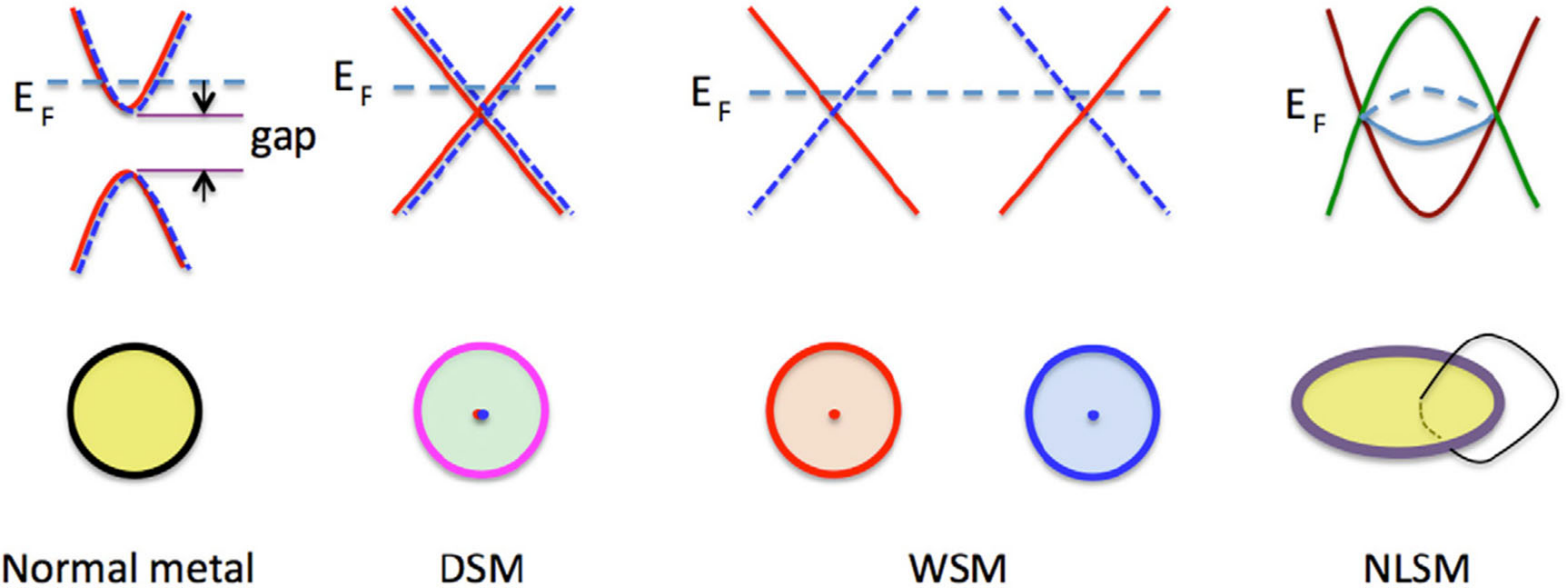
Schematic band structure and Fermi surface (with Fermi‐level shift slightly off the nodal points) for normal metal and three kinds of topological semimetals: DSM, WSM, and node‐line semimetal (NLSM). The closed path (black thin circle) interlocked with the nodal ring (thick circle) is also shown. Reproduced with permission.^[^ ^23^ ^]^ Copyright 2016, IOP Publishing.

Being inspired by the successful progress on topological insulators, scientists try to investigate whether metals can be classified with topological invariances or not. The ideal topological metals are those only composed of nodal points, which are called topological semimetals. Topological semimetals can be classified into four categories: Weyl semimetal (WSM), Dirac semimetal (DSM), nodal‐line semimetal, multidegenerate nodal‐point semimetal, and the corresponding schematic band structures are shown in **Figure** **3**.^[^ ^23^, ^24^ ^]^ Weyl fermions, the massless fermions with chirality predicted by the Weyl equation, are the quasiparticles of low‐energy excitations in WSMs.^[^ ^25^ ^]^ For WSMs, the crossing point of two nondegenerate bands at an arbitrary position of the Brillouin zone is called Weyl point and can be observed.^[^ ^26^ ^]^ A significant consequence of WSM is the existence of open Fermi arcs on its surface. Considering a WSM containing only a pair of Weyl points with opposite chirality that is separated by a vector **K** in momentum space, a Fermi arc that connects the projection of these two Weyl points will exist on surfaces whose normal vector is not parallel to **K**. Fermi arcs have been observed in angle‐resolved photoelectron spectroscopy experiments and their nonlocal property has attracted great attention.^[^ ^27^, ^28^ ^]^ WSMs can be further divided into type I (e.g., TaAs family of compounds) and type II (e.g., WTe_2_ or MoTe_2_ family of compounds).^[^ ^29^, ^30^, ^31^ ^]^ Unlike WSMs that generally do not require any symmetry, DSMs need crystalline symmetries and generically time‐reversal symmetry to protect the four‐fold degenerate Dirac points. Dirac points can be found on high‐symmetry points or high‐symmetry lines in the momentum space. However, a Dirac point can be split into a pair of Weyl points when the time‐reversal symmetry is broken and DSMs become WSMs. DSMs can also become topological insulators after breaking the crystalline symmetry and protecting it. There have been proposals that Na_3_Bi, Cd_3_As_2_, BiO_2_, and some other materials are DSMs.^[^ ^32^, ^33^, ^34^ ^]^ Na_3_Bi and Cd_3_As_2_ are the first experimental examples of DSMs and have been extensively studied. Valence and conduction bands cross at points for WSMs and DSMs, while they can cross to form nodal lines or rings to form topological nodal‐line semimetals.^[^ ^35^, ^36^ ^]^ The line nodes with four‐fold degeneracy including spin degree of freedom can be formed by the crossing of two Kramer degenerate bands with opposite mirror eigenvalues of the glide mirror operation on the Brillouin Zone boundary.^[^ ^37^ ^]^ This requires the compound having time‐reversal symmetry, inversion symmetry, and twofold screw symmetry.^[^ ^38^ ^]^ In the spin‐less case, when spin‐orbit coupling can be neglected, only time reversal and inversion symmetries are required to form double‐degenerate nodal line. There have been carbon Mackay−Terrones crystals, rare‐Earth nitrides, ZrSiS, Ca_3_P_2_, CaP_3_, and many others considered as nodal‐line semimetals.^[^ ^36^, ^39^, ^40^, ^41^ ^]^ When the nodal lines or nodal rings projected onto the surface, there are drum‐head like TSSs nested in the region circled or spanned by the projections of nodal lines. Very high carrier mobility, negative longitudinal magnetoresistance and linear nonsaturate magnetoresistance, anomalous Hall effect, and some other exotic phenomena are theoretically predicted and experimentally observed in topological semimetals.^[^ ^42^, ^43^, ^44^, ^45^, ^46^ ^]^

Scientists continue searching for more topological materials and investigating their potential practical applications. Although electronics, spintronics, optoelectronics, thermoelectrics, and phase‐change memory materials are believed to be promising application prospects of topological materials, fully understanding their properties and utilizing them still have a long way to go.^[^ ^47^, ^48^ ^]^ The application of topological materials in catalytic chemistry attracts chemists’ attention.^[^ ^49^, ^50^, ^51^ ^]^ It is based on the assumption that TSSs can provide a stable electron bath for surface reaction and the high carrier mobility is beneficial for electron transfer between surface and reaction intermediates. Compared with current catalysts, topological semimetals have excellent conductivity, which is an advantage for electrocatalysis. In addition, TSSs are robust against surface deformation or reformation (e.g., defect creation and oxidation), which is often observed during many catalytic activities. Therefore, the advantage of TSSs can persist under various environments. Recent investigations of topological catalysts are shown in the next section.

## 2 Current Progress of Topological Catalysts

To date, only a few research groups have started investigations on topological materials catalysts (TMCs) and about a dozen research articles have been published. Currently, the investigation of TMCs mainly focuses on topological semimetals, topological insulators, and TCIs. Various catalytic reactions, such as hydrogen evolution reaction (HER), oxygen evolution reaction (OER), CO_2_ reduction reaction (CO_2_RR), and ethanol oxidation reaction (EOR), are investigated and excellent catalytic performance compared with topological trivial material catalysts is shown in **Table** **1**. Though there is still no consensus on the mechanism of the high catalytic activity of TMCs, some researches make efforts to open the mystical veil of this attracting topic, as shown in the following paragraphs. The TMC papers focus on topological semimetals is more than topological insulators and TCIs. So, we will begin with topological semimetals and then topological insulators and TCIs.

**Table 1:** Comparison of the catalytic properties between current excellent catalysts and topological material catalysts for HER, OER, CO_2_RR, and EOR

Catalytic reaction	Catalyst	Topological material category	Catalytic property	References
HER	NbP	WSM	The rate of HER on NbP (87.7 μmolm^−2^ h^−1^) shows higher activity than on Pt−TiO_2_ (26.1 μmolm^−2^ h^−1^)	[52]
HER	MoS_2_/Bi_2_Te_3_/SrTiO_3_ heterostructure	Topological insulator	The existence of topological insulator Bi_2_Te_3_ reduces the overpotential from 437 (MoS_2_//SrTiO_3_) to 248 mV (MoS_2_/Bi_2_Te_3_/SrTiO_3_)	[55]
OER	Co_3_Sn_2_S_2_	WSM	The overpotential on bulk crystal Co_3_Sn_2_S_2_ (300 mV) is close to CoN nanowires (290 mV) and NiCo metal−organic framework nanosheets	[54]
CO_2_RR	Bi	TCIs	Compared with Bi nanosheet, Bi RDs exposed with (110) and (104) facets show lower overpotential (120 mV) and higher formate selectivity (FE >92.2%)	[51]
EOR	PdTe_2_	DSM	The PdTe_2_ nanosheet shows fivefold mass activity compared with commercial Pd black	[60]

### 2.1 Topological Semimetal Catalysts

**Figure 4:**
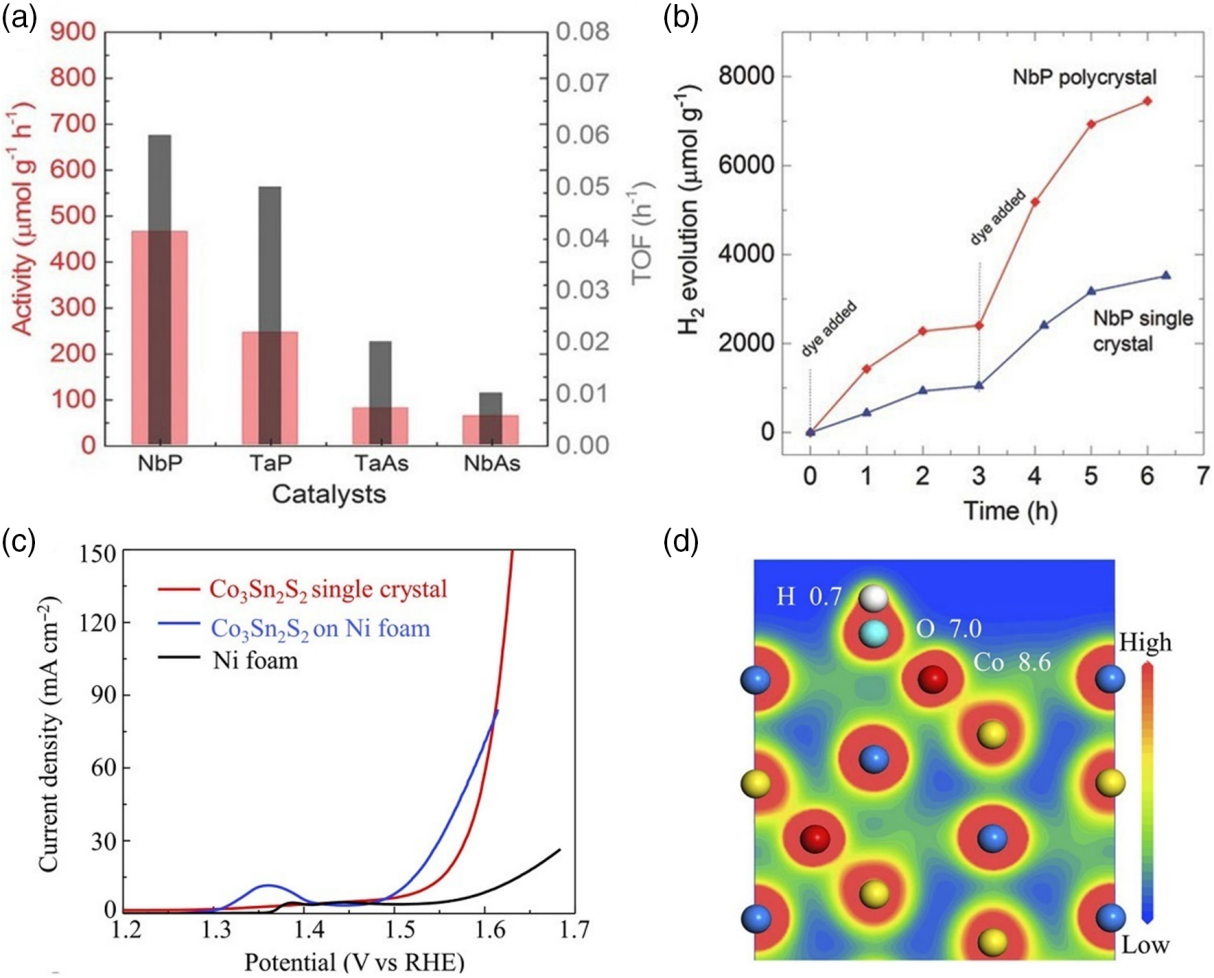
a) Histogram of hydrogen evolution rate and turn over frequency (TOF), shown on the left and right axes, respectively, for all NbP, TaP, NbAs, and TaAs. b) Comparison of H_2_ evolution of NbP in polycrystalline and single‐crystalline powder form. Polycrystalline powders show higher catalytic activity compared with NbP single crystals. Reproduced with permission.^[^ ^52^ ^]^ Copyright 2017, Wiley‐VCH. c) OER polarization curves for Ni foam, Co_3_Sn_2_S_2_ single crystal, and Co_3_Sn_2_S_2_micropowder crushed from the single crystal. d) Contour plots of the total charge distribution of Co_3_Sn_2_S_2_ single crystal with one OH group bonded to the Co atoms. Electronic charges are distributed in the vicinity of Sn atoms. However, for Co atoms, one can see the electron transfer through the Co—O bonding. Reproduced with permission.^[^ ^54^ ^]^ Copyright 2019, American Association for the Advancement of Science.

As the first family of topological WSMs, transition metal monopnictides (NbP, TaP, NbAs, and TaAs) were studied as photocatalysts for HER. This is motivated by the idea that the combination of robust TSSs and large‐room‐temperature carrier mobility of WSMs can enhance catalytic activities.^[^ ^52^ ^]^ As shown in **Figure** **4**a, all the four WSMs are highly active and NbP is the best. Phosphides are on average better than arsenides. Chemical analysis shows that catalysts are stable after several HER cycles. A twofold increase of the activity is observed in polycrystalline NbP (150–300 nm in size) compared with the NbP single crystals crushed into powder (few micrometers in size), which has a smaller surface area (Figure 4b). Thus, an increased surface area can lead to a higher catalytic activity. Compared with topologically trivial materials, the authors claimed that size reduction plays a more important role in topologically nontrivial materials because nontrivial TSSs are protected by topology. Therefore, the HER activities of WSMs can be enhanced by reducing the size of the catalysts and the negative effect of crystal disorder will be minimized. In the same year, Chen et al. performed theoretical calculations on the topological Dirac nodal‐line (DNL) semimetals (TiSi family semimetals) for HER, which is inspired by the idea that extremely high electronic density near the Fermi level comes from the TSSs of topological DNL semimetals which may lead to a high HER performance. The results indicate that TiSi family (TiSi, TiGe, and TiSn) DNL semimetals are promising HER catalysts, and the topological charge carriers are involved in the catalytic process.^[^ ^53^ ^]^ In addition, Co_3_Sn_2_S_2_ is the first magnetic WSM, which was investigated to show high performance for OER.^[^ ^54^ ^]^ As shown in Figure 4c, low overpotential of 300 mV is needed to reach a current density of 10 mA cm^−2^ for bulk single‐crystal Co_3_Sn_2_S_2_, when the reference is set to the thermodynamic OER potential (*E*_0_ = 1.23 V vs. RHE). This overpotential is close to or even lower than those nanostructured electrocatalysts, such as (Ni/Co)_0.85_Se nanotube arrays, CoN nanowires, and NiCo metal−organic framework nanosheets. The OER performance can be further enhanced with an overpotential of 270 mV after crushing the bulk single crystal of Co_3_Sn_2_S_2_ into particles and depositing them on Ni foam. Therefore, a larger surface area can lead to better performance for OER, which is similar to HER on NbP. The exposed surface of the sample is (001) surface with a termination of a layer of Co. As the crucial step of OER, the initial discharge of hydroxide ions at the active center is investigated. The surface Co‐3*d* states split into one doubly degenerate state and three nondegenerate states (including half‐filled 3*d*_*z*_^2^ states). The bonding character between exposed Co atom and *OH intermediate is vividly shown in Figure 4d. The half‐filled 3*d*_*z*_^2^ orbital points toward the *p* orbital of the adsorbed hydroxide ions and the dumbbell‐shaped charge distribution on Co atoms indicate a 3*d*_*z*_^2^ orbital involved in the adsorption process. It can be seen that electrons transferred from the *OH intermediate to the surface Co atom through Co—O bonding as the total charge on Co and *OH is 8.75 and 7.67 |*e*|, respectively. Theoretical calculations and angle‐resolved photoelectron spectroscopy measurements confirm that the surface states derived by Co are topologically protected, which guarantees the existence of these surface states not being destroyed during the reaction process unlike other active sites (e.g., defects and doping). Therefore, the surface Co atoms are robust active sites for OER.

### 2.2 Topological Insulator Catalysts

**Figure 5:**
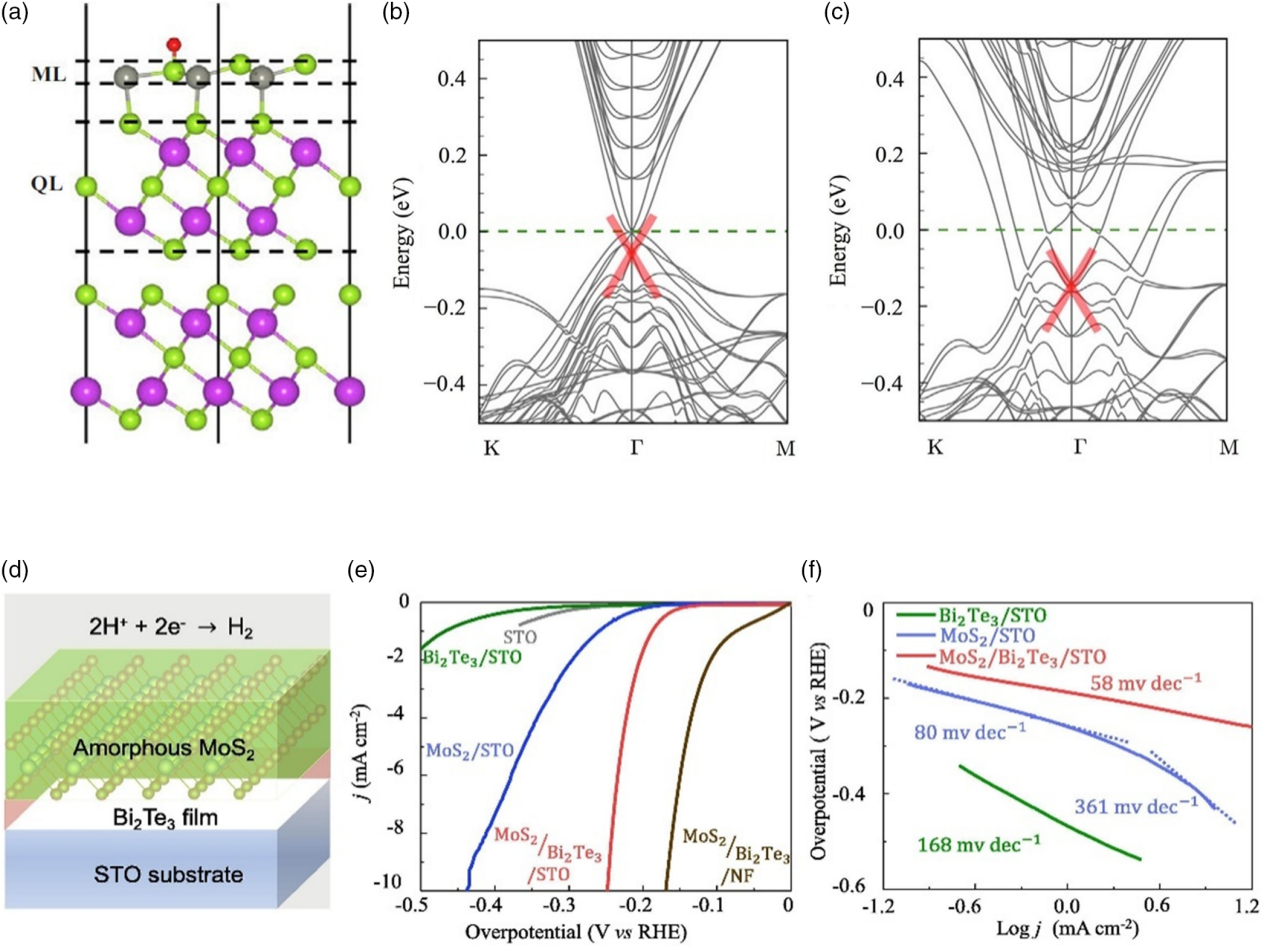
a) Side view of the structure for 1‐ML ZnSe/Bi_2_Se_3_. The red, green, gray, and purple balls represent H, Se, Zn, and Bi atoms, respectively. Band structures of 1‐ML ZnSe/Bi_2_Se_3_ b) without and c) with hydrogen adsorption, respectively. The upper‐surface TSS bands around the Γ point are highlighted by the red thick lines. Reproduced with permission.^[^ ^50^ ^]^ Copyright 2019, Elsevier. d) Illustration of the MoS_2_/Bi_2_Te_3_/STO heterostructure catalysts. e) HER polarization curves of the pure STO substrate, MoS_2_/STO, Bi_2_Te_3_/STO, and MoS_2_/Bi_2_Te_3_/STO catalysts. f) Corresponding Tafel plots were obtained from the polarization curves in (e). Reproduced with permission.^[^ ^55^ ^]^ Copyright 2021, Elsevier.

Although a lot of topological materials have been discovered since the first experimental verification of HgTe/CdTe quantum wells, only few of them are experimentally tested as catalysts. Among them, Bi_2_Se_3_ is a typical topological insulator, whose TSSs are demonstrated to be effective electron baths for enhancing the adsorption of CO and O_2_ when Bi_2_Se_3_ is covered by gold.^[^ ^49^ ^]^ Thus, it is reasonable to expect to tune the adsorption strength of reaction intermediates to enhance the catalytic activity. Forming heterostructures by covering Bi_2_Se_3_ with topologically trivial materials is a promising way to get excellent catalysts. For instance, density functional theory (DFT) calculations found that heterostructures of Bi_2_Se_3_ covered with a single layer of ZnSe is an ideal platform for HER.^[^ ^50^ ^]^ The *H adsorption free energy, ΔG_*H_, is a reasonable descriptor of hydrogen evolution activity on HER catalysts. The ideal HER catalyst should have a moderate ΔG_*H_ which is close to 0 eV. The adsorption of *H on the pristine Bi_2_Se_3_ (0001) surface is too weak (ΔG_*H_ > 1.0 eV), while too strong on ZnSe (111) surface (Δ*G* < −0.7 eV). For Bi_2_Se_3_ covered with a single‐layer ZnSe (1‐ML ZnSe/Bi_2_Se_3_, **Figure** **5**a), ΔG_*H_ is 0.21 eV without considering spin orbit coupling (SOC) and decreases to −0.02 eV with SOC, which means that the topological effects of TSSs on Bi_2_Se_3_ can enhance the adsorption. Therefore, 1 ML ZnSe/Bi_2_Se_3_ is a promising catalyst for HER. Band structures indicate that there is a direct interaction between TSSs and *H intermediates due to the downshift in energy level of the TSSs after the adsorption of *H, as shown in Figure 5b,c. The adsorption of *H becomes too strong for heterostructures with multiple layers of ZnSe because the effect of TTSs from Bi_2_Se_3_ is almost negligible. Inspired by the earlier theoretical study, the experiment of MoS_2_/Bi_2_Te_3_/STO (STO for SrTiO_3_) catalyst is designed and the heterostructure is illustrated in Figure 5d, where Bi_2_Te_3_ is a topological insulator, while MoS_2_ and STO are topologically trivial materials.^[^ ^55^ ^]^ As shown in Figure 5e, pure STO and Bi_2_Te_3_/STO nearly do not show HER activities. MoS_2_/STO presents enhanced HER efficiency with an overpotential of 437 mV to reach a current density of 10 mA cm^−2^. In contrast, an overpotential of only 248 mV was required to reach the same current density of 10 mA cm^−2^ for MoS_2_/Bi_2_Te_3_/STO. A similar topological effect as described in the earlier theoretical study exists on the top layer of MoS_2_, which comes from Bi_2_Te_3_. Thus, introducing nontrivial TSSs is an effective way to manipulate the HER activity.

### 2.3 TCI Catalysts

**Figure 6:**
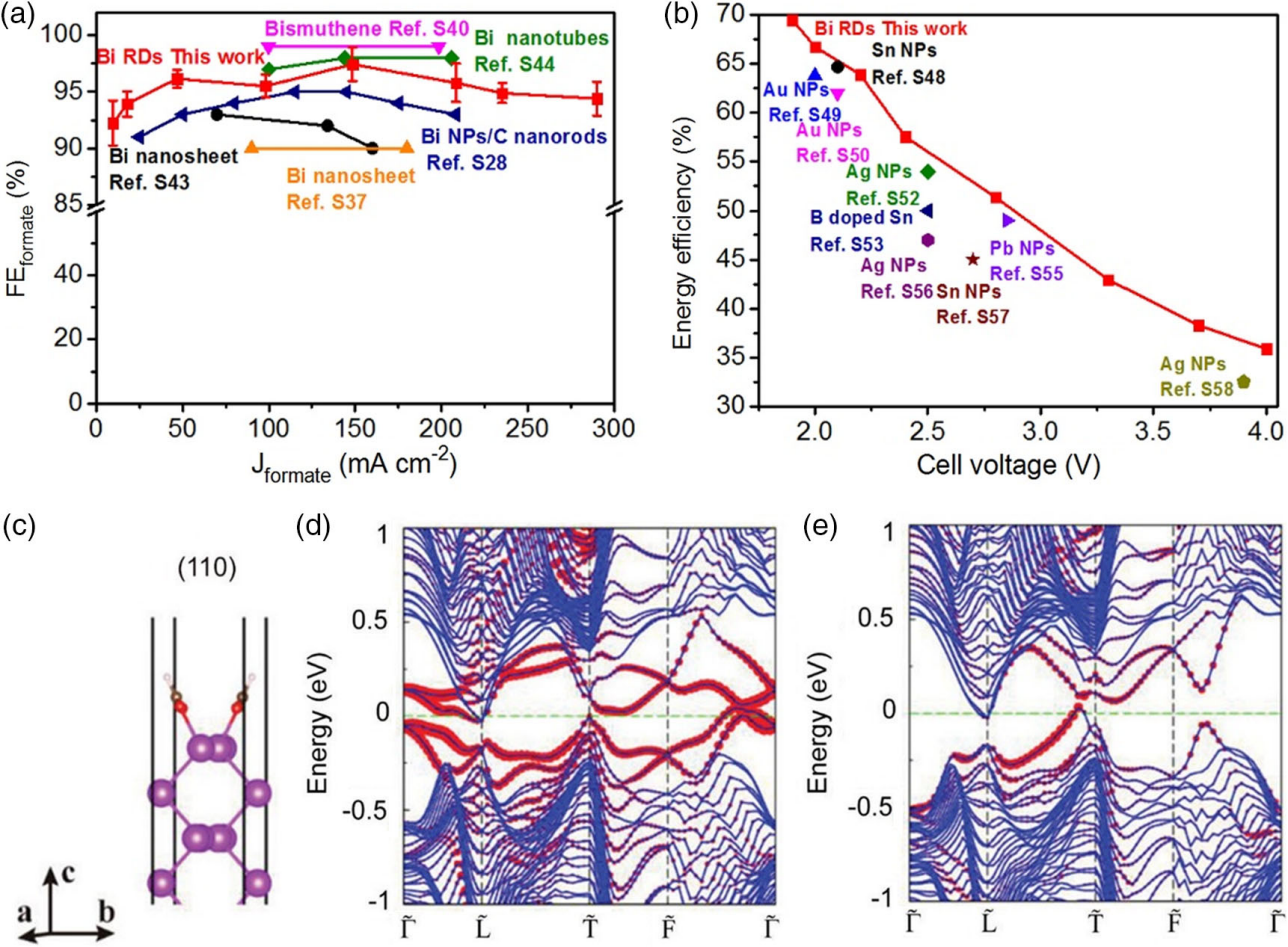
a) Comparison of the partial current density range for highly selective formate production (FE_formate_ > 90%) on C‐Bi RDs and the reported Bi‐based electrocatalysts. b) Energy efficiencies of formate production on C‐Bi RDs at different cell voltages and those of the electrocatalysts reported in the literatures (NPs: nanoparticles). c) Structures of the Bi (110) surface with the adsorption of *OCHO intermediate. The purple, red, brown, and pink balls represent Bi, O, C, and H atoms, respectively. d,e) The surface states of (110) surface without (b) and with (c) the adsorption of *OCHO intermediate. The high‐symmetry points are $\tilde{\text{Γ}}$(0, 0),$\tilde{\text{ L}}$ (0.5, 0), $\tilde{T}$(0.5, 0.5), and $\tilde{F}$ (0, 0.5) in fractional coordinates and the red dots represent the projections of Bi orbitals on the surface. Reproduced with permission.^[^ ^51^ ^]^ Copyright 2021, Wiley‐VCH.

For ordinary topological materials such as topological insulators and topological semimetals, the topological properties are independent of size and shape. However, TCI is a special type of topological material, where TSSs are strongly dependent on the morphology and only exist on the surfaces preserving the crystalline symmetry required to protect the bulk band topology. This means that researchers can design the shape of TCI samples to have different surfaces with and without TSSs and test them simultaneously in the same experimental condition. Comparing the catalytic performance of different TCI surfaces would facilitate to clarify the mechanisms related with TSSs. Bismuth (Bi) is an efficient electrochemical catalyst for CO_2_RR (ECO_2_RR) to produce formate. At the same time, Bi is a TCI with gapless TSSs protected by a specific crystalline symmetry that strongly depends on the surfaces. These make Bi the first investigated TCI catalyst. It was recently reported that TSSs on Bi (110) is the key factor where Bi has high selectivity, activity, and full‐cell energy efficiency for ECO_2_RR.^[^ ^51^ ^]^ Single‐crystalline Bi rhombic dodecahedrons (RDs) exposed with plenty of (104) and (110) facets were synthesized. It was found that formate can be detected at −0.37 V versus reversible hydrogen electrode (RHE) with a Faradic efficiency (FE) of 89.6% and the overpotential is as low as 120 mV. The FEs of formate exceed 92.2% on Bi RDs within the potential range of −0.42−−0.78 V versus RHE. The maximum current density changes in a very wide range, 9.8–290.1 mA cm^−2^ (**Figure** **6**a), which indicates the outstanding selectivity toward formate. In addition, the full‐cell energy efficiency of formate production is remarkably high, of 69.5% at 1.9 V, which is among the highest of the reported electrocatalysts for ECO_2_RR (Figure 6b). Theoretical calculations reveal that the nontrivial TSSs on Bi (110) keep existing after the adsorption of *OCHO, while the trivial surface states disappear, as shown in Figure 6d,e. As TSSs would not be affected by applied potential changes, they enhance the adsorption of *OCHO, which is beneficial for efficient ECO_2_RR.^[^ ^56^ ^]^ For Bi (104) surface, small gaps after the adsorption of *OCHO guarantee the high mobility of charge transfer between the catalyst and *OCHO intermediates. The surface states on Bi (012) surface are trivial and unstable, which will be destroyed after the adsorption of *CHOH. Therefore, Bi (110) and (104) surfaces are active for ECO_2_RR due to the existence of sufficient surface states. Especially, the nontrivial TSSs on the Bi (110) surface provide a stable electron bath for a high rate and energy‐efficient ECO_2_RR. the shape of Bi RDs is unchanged after the reaction, which guarantees the persistence of TSSs during the catalytic process. This study confirms the link between TSSs and catalytic activity in a more cogent way because that facets with TSSs are more likely to show better performance than topological trivial surfaces in a same catalyst. It opens a new way to investigate the TSSs‐related mechanisms for TMCs.

Other topological materials also show outstanding catalytic activities besides systems presented earlier, such as the TiSi‐type family of nodal‐line semimetals,^[^ ^53^, ^57^ ^]^ Dirac nodal‐arc semimetal PtSn_4,_^[^ ^58^ ^]^ chiral crystals for electrochemical HER^[^ ^59^ ^]^, type‐II DSM PdTe_2_ for ethanol electro‐oxidation,^[^ ^60^ ^]^ and so on.^[^ ^61^, ^62^, ^63^, ^64^, ^65^, ^66^ ^]^ There are still many basic problems unclear though some progresses have made in topological catalysts, such as the concrete role that TSSs play in high catalytic performance, the persistence of TSSs under acid or alkaline reaction environment, etc.

## 3 Perspective

Though recent studies have shown that topological materials are new classes of catalysts with many advantages, many fundamental works are still needed to be done in the future to gain better knowledge on them. Appropriate binding energy of the reaction intermediates on the catalyst surface is essential for an efficient catalytic activity. Take CO_2_RR for instance, a too weak adsorption of *CO intermediates will cause the release of CO gas instead of producing more value‐added chemicals (e.g., C_2_H_4_), while too strong adsorption of reaction products will make the surface be covered by these products and inhibit following reactions. Thus, it is important to understand the interaction between the intermediates and surface states of the catalysts. Both trivial surface states from dangling bonds and TSSs participate in the adsorption process at the same time, which brings difficulties to distinguish their individual effects. However, revealing the origin of the outstanding performance of TMCs heavily relies on a thorough understanding of how TSSs affect the adsorption of intermediates. On the one hand, surface‐dependent TSSs such as TCI is an idea platform to design contrast experiments to figure out the exact role that TSSs play in the enhanced catalytic performance. TSSs in TCI are protected by a specific crystalline symmetry, which makes it possible to test the catalytic activity on facets with or without TSSs for samples in different morphologies. There should be two different surfaces with the same amount of dangling bonds, while one surface has TSSs and another does not. It is reasonable to expect to reveal that the effect comes from TSSs by comparing these two surfaces. On the other hand, topological materials exposed with the same facet but different atomic terminations have different amounts of dangling bonds (e.g., Bi_2_Se_3_(0001) can be terminated with Bi or Se),^[^ ^49^, ^67^ ^]^ which can also be used to investigate the characteristic function of TSSs. Ab initio calculations should be applied to this investigation because they can offer underlying details in a convenient and economic way, such as forming samples with different shapes, manipulating the exposed terminations, and analyzing the bonding characters.

Although TSSs are robust against local perturbations such as doping, defect creation, chemisorption of reaction intermediates, and slight oxidation when exposed under the ambient environment, the existence of TSSs during the catalytic reaction has to be made sure. It is because the deformation or reformation of surface during the catalysis process is often observed due to oxidation, the high coverage of intermediates, and strong acid or strong base of the reaction environment. Severe surface deformation or reformation will have an impact on the topological electronic properties and then the catalytic activity. Take TCI SnTe for instance; defects that break local symmetry can obstruct TSSs, as shown by a threefold reduction of the spectral weight of the TSSs.^[^ ^68^ ^]^ Angle‐resolved photoelectron spectroscopy and ab initio calculations can be used to determine whether TSSs still exist after being affected by the catalytic reaction process. Long existing TSSs allow topological catalysis to be efficient and durable. Avoiding the suppression of TSSs and choosing a suitable working environment can make the most of the advantages of TSSs.

Making heterostructures by covering topological materials with topologically trivial films is a promising way to tune the adsorption energy for those topological materials binding intermediates too strongly or too weakly.^[^ ^50^, ^55^ ^]^ By doing this, it is possible to have an appropriate binding energy and make use of TSSs at the same time as TSSs can float to the top of the film.^[^ ^69^ ^]^ Doping other elements or creating intrinsic defects in the surface of catalysts without destroying TSSs can also tune the adsorption energy. For example, the selection of dopants for TCIs can be extended to other elements as the topology is protected by crystal symmetry. Therefore, pristine topological materials with low catalytic activities also have chance, turning into good catalysts. Currently, only few numbers of TMCs are investigated and limited reactions such as HER, OER, and CO_2_RR are focused on. So, more TMCs are needed to be discovered, and more catalytic reactions are to be studied for TMCs in the future. It is suggested to search candidates by means of ab initio calculations and then verify them by experiments which will save time and resources.

The progresses, advantages, and challenges for topological materials in catalysis are presented in this Perspective. We believe that this new emerging research field will attract more attention and bring breakthroughs in future.

## Conflict of Interest

The authors declare no conflict of interest.
